# Mind–Body Training: A Plausible Strategy against Osteomuscular Chronic Pain—A Systematic Review with Meta-Analysis

**DOI:** 10.3390/jpm14020200

**Published:** 2024-02-11

**Authors:** Julia Gámez-Iruela, Agustín Aibar-Almazán, Diego Fernando Afanador-Restrepo, Yolanda Castellote-Caballero, Fidel Hita-Contreras, María del Carmen Carcelén-Fraile, Ana María González-Martín

**Affiliations:** 1 Department of Health Sciences, Faculty of Health Sciences, University of Jaén, 23071 Jaén, Spain; 2 Faculty of Health Sciences and Sport, University Foundation of the Área Andina-Pereira, Pereira 660004, Colombia; 3 Department of Education and Psychology, Faculty of Social Sciences, University of Atlántico Medio, 35017 Las Palmas de Gran Canaria, Spainana.gonzalez@atlanticomedio.es (A.M.G.-M.); 4 Department of Psychology, Centro de Educación Superior de Enseñanza e Investigación Educativa, Plaza de San Martín, 4, 28013 Madrid, Spain

**Keywords:** chronic pain, mind–body exercises, older adults, systematic review, meta-analysis

## Abstract

(1) Background: Chronic pain, which affects more than one in five adults worldwide, has a negative impact on the quality of life, limiting daily activities and generating absences from work. The aim of the present review is to analyze the efficacy of mind–body therapies as therapeutic strategies for patients with chronic pain. (2) Methods: A systematic review with a meta-analysis was carried out, searching PubMed, Scopus, and Web of Science databases using specific keywords. We selected studies that included mind–body therapies as the primary intervention for older adults with chronic pain. The methodological quality of the articles was assessed using the PEDro scale. (3) Results: Of the 861 studies identified, 11 were included in this review, all of which employed different mind–body therapies as an intervention. The selected studies measured chronic pain as the main variable. (4) Conclusions: This review highlights the value of mind–body exercises in reducing chronic pain in older adults, suggesting their integration as a non-pharmacological therapeutic alternative that improves the quality of life, promoting a holistic approach to pain management.

## 1. Introduction

Patients seeking medical guidance most frequently mention pain as a major symptom, which is one of the leading causes of disability globally [1]. Pain is described as ‘an unpleasant sensory and emotional experience associated with or resembling that associated with actual or potential tissue damage’ [2]. Chronic pain (CP) is defined as pain that lasts or recurs for more than three months, regardless of whether it is experienced in one or more anatomical regions [3]. It is estimated that CP affects more than one in five adults [4], and the global prevalence of chronic pain affects more than 30% of the world’s population [5]. Additionally, it has been shown that this prevalence increases with age, and the negative impact of pain tends to be greater among older adults compared to younger ones [6]. This is particularly notable in subjects with cardiovascular disease, smokers, the female gender, and patients with depressive disorders [7]. In certain circumstances, chronic pain is related to other diseases as an underlying cause, i.e., secondary chronic pain, such as in cancer and postoperative, post-traumatic and neuropathic pain, among others [3]. Meanwhile, in other cases, it constitutes a condition of its own, i.e., mainly chronic pain: fibromyalgia, migraines, irritable bowel syndrome and lower back pain, among others [2]. It has been found that those with chronic pain experience a marked reduction in their quality of life, facing limitations in their daily activities, including social interactions and basic tasks [8]. Also, patients with chronic pain are more likely to be absent from work compared to those who do not experience chronic pain [9].

Typically, pharmacological methods, such as analgesics, are the first option prescribed to treat chronic pain, aiming to reduce the intensity of symptoms and promote normal development [10]. Within this pharmacological approach, opioids have been widely used, offering significant relief for severe pain. However, their widespread use has led to an unprecedented public health crisis, marked by the exponential increase in cases of dependence, tolerance and, most alarmingly, fatal overdoses [11]. This situation has highlighted the need to re-evaluate the use of opioids in the management of chronic pain, which is why safe and effective alternatives are being sought to prevent the risks associated with their prolonged use that compromise the quality of life of patients [12]. Therefore, the current challenge is to develop therapeutic strategies that not only mitigate chronic pain but also promote improved physical function and emotional well-being, thereby reducing the overall burden of chronic pain and directly addressing the complexities arising from problematic opioid use [13]. Studies have indicated that the practice of physical activity and the use of cognitive behavioral therapy play significant roles in the management of chronic pain, improving the quality of life and overall well-being of individuals [14]. Specifically, properly administered exercise can have analgesic effects in the management of chronic pain and is considered a critical non-drug component [15]. It has been noted that the non-pharmacologic approach to pain management involves behavioral, cognitive, integrative and physical therapies, promoting tissue recovery and the restoration of functional mobility [16].

Within the field of physical exercise, mind–body interventions (MBIs) stand out as an essential component for improving the quality of life in people experiencing chronic pain [17]. An MBI is a mild- to moderate-intensity physical activity that, according to the National Center for Complementary and Integrative Health (NCCIH), encompasses a variety of methods and techniques focusing on the inter-relationships between mind, body and behavior and their influence on health [18]. Disciplines such as Tai Chi (TC), yoga and Qigong (e.g., Baduanjin and Wuqinxi) represent the three most widespread methods of mind–body exercise (MBE). These involve a diversity of movements ranging from stretching postures to skeletal muscle relaxation, as well as breath control and a meditative state of mind [19].

These approaches have been used to treat various chronic pain conditions and are considered appropriate for the elderly [20]. It is also relevant to mention that the appropriate use of mind–body methods (MBMs) carries minimal or no risk of side effects, making them very attractive therapeutic options [21]. Despite this, very few reviews have paid attention to the role of mind–body exercise (MBE) in the treatment of chronic pain in adults [22,23,24,25], and most of these studies have focused on a single chronic pain condition [24,25] or investigated a single MBE method [21,22]. Therefore, this systematic review and meta-analysis aims to analyze the efficiency of mind–body therapies as therapeutic strategies for patients with chronic pain.

## 2. Materials and Methods

This systematic review aims to evaluate the effectiveness of mind–body training interventions in reducing pain among patients with chronic musculoskeletal pain. For the development of the review, we considered the protocols outlined in the 2020 PRISMA declaration [26] and the pre-specified protocol registered in PROSPERO (CRD42024497802). Additionally, we adhered to the methodological recommendations presented in the ‘Cochrane Manual for the Elaboration of Systematic Reviews of Interventions’ [27].

### 2.1. Sources of Information

The bibliographic search was conducted between October and December 2023 in the PubMed, Scopus, and Web of Science (WOS) databases.

### 2.2. Search Strategy

Different keywords were used in the following search string: (‘persistent pain’ OR ‘chronic pain’ OR ‘Pain’) AND (‘Pilates’ OR ‘Yoga’ OR ‘Tai Chi’ OR ‘Core-Based’ OR ‘Mind-Body’) AND (‘older adults’ OR ‘elderly’ OR ‘seniors’ OR ‘aging’).

### 2.3. Inclusion Criteria

The included articles had to satisfy the following inclusion criteria: (i) being randomized clinical trials (RCTs), (ii) encompassing interventions grounded in mind–body training for individuals experiencing pain due to musculoskeletal diseases, and (iii) specifying the duration of subjects’ pain, which had to be equal to or greater than 3 months.

### 2.4. Exclusion Criteria

We excluded studies in which there was the presence of other conditions such as cancer; a cerebrovascular accident (CVA); and cardiovascular (CVD), pulmonary, and/or renal disease. Studies involving patients with chronic pain unrelated to the musculoskeletal system were excluded. Additionally, articles belonging to the gray literature were excluded from this review.

### 2.5. Study Selection Process

The initial screening involved the elimination of duplicate articles and those without available abstracts. Subsequently, titles and abstracts were thoroughly reviewed to exclude articles that did not align with the previously specified eligibility criteria. Finally, full-text articles underwent examination to confirm whether they met the inclusion criteria. The screening process was carried out independently by two authors (A.A.-A and J.G.-I.). Any discrepancies were resolved through consensus with a third author (Y.C.-C.). Data extraction encompassed various elements, including authors, year of publication, location, population details (sample size, age, and group distribution), study design, outcomes, measurement tools employed, description of intervention procedures, measurement time points, attrition rates, adverse effects, and main findings.

### 2.6. Data Extraction

The primary variables in this review were pain in patients with chronic musculoskeletal conditions. Additionally, the meta-analysis included the pathology causing the pain, the measurement instrument used for pain assessment, and the intervention as grouping variables for subgroup analyses.

### 2.7. Assessment of Methodological Quality

The methodological quality was assessed using the PEDro scale [28], an 11-item checklist. The maximum achievable score is 10 points, with the first item (‘eligibility criteria’) excluded from the final score calculation. Responses to each item are categorized as either ‘Yes’ (1 point) or ‘No’ (0 points). Scores from 0 to 3 are classified as ‘Poor’ quality, 4–5 as ‘Fair’, 6–8 as ‘Good’, and >9 as ‘Excellent’ [29].

### 2.8. Analytic Decisions for Meta-Analysis

The meta-analysis’ findings are presented in a forest plot, which includes key information such as the lead author, publication date, sample size, and individual effects measured using the Hedge index (g) with a 95% confidence interval, along with the associated *p*-value. To ensure robustness, a sensitivity analysis was conducted, excluding studies with duplicate data, values, and individual cases. The outcomes from this analysis were then compared with those obtained from the complete meta-analysis.

For stratified or subgroup analyses, studies were grouped based on pathology, the intervention, and the pain measurement instrument. Separate meta-analyses were performed within each subgroup, allowing for the exploration of variability and effect size specific to each category. This approach enhances the granularity of understanding the results. Finally, to assess the risk of publication bias, a funnel plot was employed. This analysis provides a visual representation of the distribution of study outcomes and helps evaluate the potential influence of publication bias on the overall findings.

## 3. Results

### 3.1. Studies Selection Process

**Figure 1:**
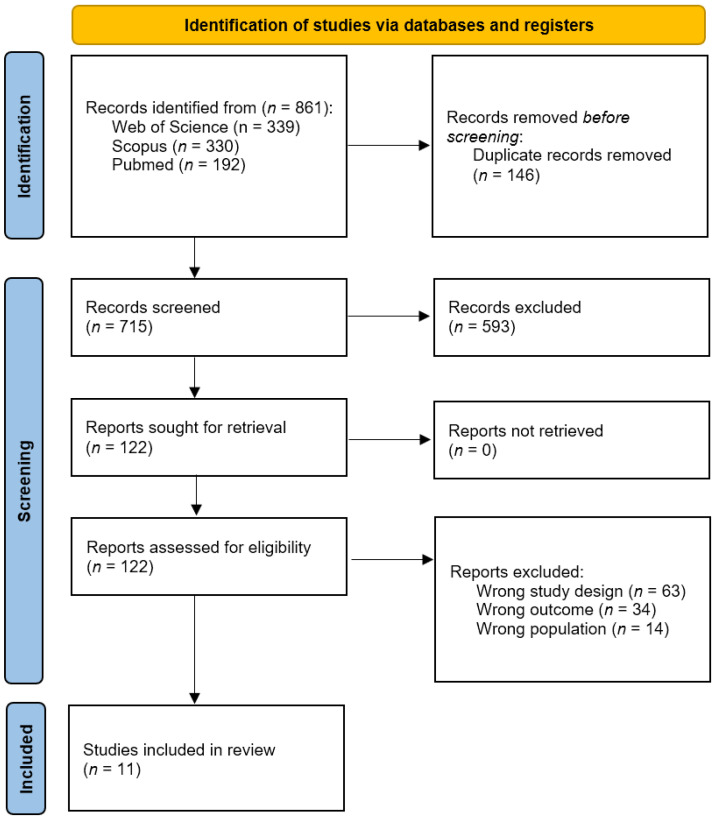
Study selection process flow chart.

The initial search of various databases revealed a total of 861 articles. Next, duplicate elimination was performed in these databases, resulting in 715 unique articles. These 715 articles were then subjected to a title and abstract review, identifying 122 as candidates for qualitative evaluation. Finally, 11 articles [30,31,32,33,34,35,36,37,38,39,40] were selected and used for this systematic review with a meta-analysis. Figure 1 presents the selection process in more detail.

### 3.2. Methological Quality

The methodological quality of the included studies was assessed using the PEDro scale. The scores of all the studies [30,31,32,33,34,35,36,37,38,39,40] were obtained from the PEDro web portal. Among the included studies, eight were rated as ‘Good’ [31,32,33,35,36,38,39,40], while only three were rated as ‘Fair’ [30,34,37]. It is important to highlight that none of the studies blinded participants or therapists. The complete methodological quality assessment can be seen in Table 1.

### 3.3. Characteristics of the Studies

All the articles included in this systematic review with meta-analysis were randomized controlled clinical trials conducted in the United States [31,32,38,39,40], Germany [35,37], Australia [36], Spain [33], Korea [34], and China [30]. A total of 951 people participated in the studies included in this review, with 433 assigned to the control group, while 518 received an intervention based on mind–body training. The prevalent sex was female in all the included studies; in the case of Cruz-Diaz et al. [33] and Cheung et al. [38], 100% of the population was female. Finally, the mean age was 71.51 ± 6.16 (Table 2).

### 3.4. Effects of Mind–Body Training on Pain

The present systematic review with meta-analysis focuses on pain as the main variable. Among the included studies, three evaluated pain using the Visual Analogue Scale (VAS) [30,34,37], while three used the Western Ontario and McMaster (WOMAC) scale. Two studies employed the Numeric Pain Rating Scale (NRS) [32,33], another utilized the Short-form McGill Pain Questionnaire (MPQ-SF) [31], and a different study employed the Brief Pain Inventory (BPI) [40]. Finally, Teut et al. [35] established five levels of pain intensity and asked participants to choose how they had felt in the last 7 days. Among the 11 included studies, four [31,32,35,37] reported no statistically significant changes in patient pain.

**Figure 2:**
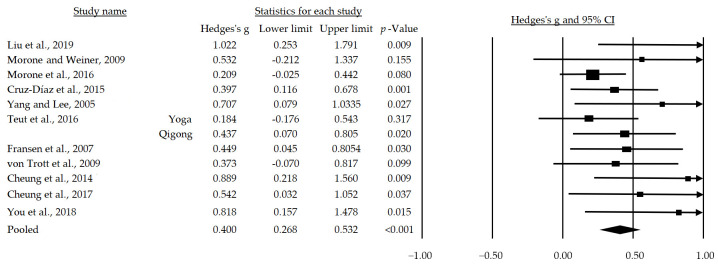
Forest plot of the overall effect of mind–body training on chronic pain [30,31,32,33,34,35,36,37,38,39,40].

The meta-analysis considered the inclusion of 11 articles. In the case of Teut et al. [35], each intervention group (the Yoga group and the Qigong group) was independently analyzed. The random effects model was employed in this meta-analysis due to low heterogeneity and variability (I2 = 10%; Q-Value = 12.182 with 11 degrees of freedom and *p* = 0.350). Following the meta-analysis, a small yet statistically significant mean effect size of mind–body interventions on pain in patients with chronic musculoskeletal pain was observed (g = 0.400, 95%CI = 0.268–0.532; *p* < 0.001) (Figure 2).

### 3.5. Subgroup Analysis

#### 3.5.1. Based on Pathology

**Figure 3:**
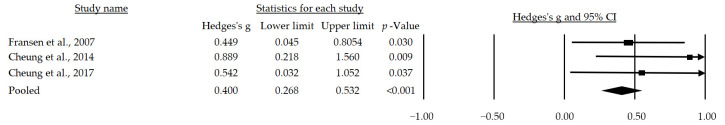
Forest plot of the pooled effect of mind–body training on chronic pain in individuals with knee osteoarthritis [36,38,39].

When conducting a subgroup analysis with the type of chronic pain as the grouping variable among research subjects, it is evident that mind–body training yields a larger effect size in knee osteoarthritis (g = 0.559, 95%CI = 0.272–0.846; *p* < 0.001) compared to cases of lower back chronic pain (g = 0.306, 95%CI = 0.145–0.468; *p* < 0.001). Nevertheless, in both scenarios, the effect size is statistically significant (Figure 3).

#### 3.5.2. Based on the Intervention

**Figure 4:**
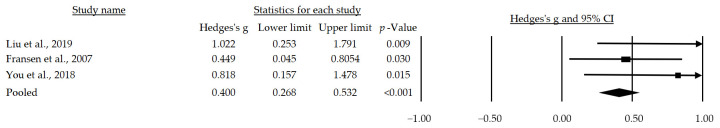
Forest plot of the pooled effect of Tai Chi intervention on chronic pain [30,36,40].

In analyzing the interventions utilized, the meta-analysis revealed that Tai Chi yielded a larger effect size (g = 0.635, 95%CI = 0.311–0.959; *p* < 0.001) compared to yoga (g = 0.462, 95%CI = 0.070–0.855; *p* = 0.021) or Qigong (g = 0.461 95%CI = 0.203–0.719; *p* < 0.001) (Figure 4).

#### 3.5.3. Based on the Pain Measurement Instrument

**Figure 5:**
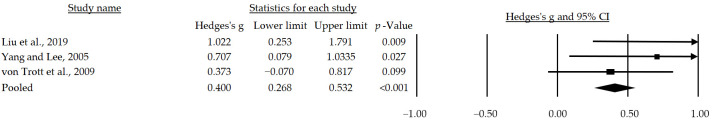
Forest plot of the pooled effect of mind–body training on chronic pain assessed with VAS [30,34,37].

In the case of instruments used to assess pain, both the VAS (g = 0.597, 95%CI = 0.242–0.952; *p* = 0.001) and the WOMAC (g = 0.559, 95%CI = 0.272–0.846; *p* < 0.001) exhibited similar effect sizes, both of which were statistically significant. Additionally, a statistically significant small effect size was observed with the NRS (g = 0.227, 95%CI = 0.026–0.429; *p* = 0.027) (Figure 5).

### 3.6. Publication Bias

Upon a thorough examination of the Funnel plot, it is evident from the symmetrical distribution of the graph that any concern regarding publication bias can be effectively dismissed.

## 4. Discussion

The present systematic review with meta-analysis included a total of 11 selected articles [30,31,32,33,34,35,36,37,38,39,40], and its main objective was to analyze the scientific literature on the effects of mind–body exercises for chronic pain in older adults. The findings revealed that the groups that performed different interventions based on mind–body therapies showed better results in terms of osteo-muscular chronic pain compared to a control group.

Regarding the methodological quality of the studies, most of the articles reviewed [31,32,33,35,36,38,39,40] obtained good methodological quality, and three articles [30,34,37] were rated as acceptable. It is relevant to note that none of the studies obtained a rating of excellent quality. It is important to mention that the lack of blinding in both patients and therapists, as well as poor task assignment, were identified as common shortcomings in these studies, which possibly influenced the results obtained. The scientific literature has indicated that the lack of blinding in patients and therapists, together with inadequate assignment, could contribute to increases of 13% and 7%, respectively, in the exaggeration of results [41].

The main variable of the selected studies is chronic pain, a complex experience that lasts for prolonged periods or recurs and represents a significant challenge to the health and well-being of millions of people around the world [42]. This type of pain affects not only the physical but also the emotional, social, and cognitive dimensions of those who suffer from it [43]. From musculoskeletal conditions to neuropathic diseases, chronic pain manifests itself in various forms, challenging the quality of life and daily functionality of those who experience it. Therefore, it is essential to use a series of instruments and/or tools to assess it [44]. The findings of this review show an interesting diversity in the instruments used to measure pain in the studies included in the systematic review and meta-analysis. Variability in pain assessment tools can be both a strength and a limitation in this type of analysis [45]. On the one hand, the use of multiple scales to assess pain, such as the Visual Analogue Scale (VAS), the Western Ontario and McMaster (WOMAC) scale, the Numerical Pain Rating Scale (NRS), the McGill Abbreviated Pain Questionnaire (MPQ-SF), the Brief Pain Inventory (BPI), and Teut et al.’s [35] categorization into five levels of pain intensity, provides a more comprehensive and diverse view of participants’ pain experience. This may offer a more holistic understanding of the effects of interventions on chronic musculoskeletal pain, considering different aspects of its intensity, localization, and sensory qualities [46].

However, this diversity of instruments may make direct comparisons between studies difficult. Each scale may capture unique aspects of pain, making it challenging to aggregate data for a pooled analysis, thus affecting the accuracy and overall interpretation of meta-analysis results [47]. This aspect highlights the need for the careful consideration of pain measurement instrument selection in future research and underscores the importance of standardizing assessments to facilitate comparisons across studies and strengthen the validity of meta-analyses.

It is noteworthy that the WOMAC (g = 0.559, 95%CI = 0.272–0.846; *p* < 0.001) and the VAS (g = 0.597, 95%CI = 0.242–0.952; *p* = 0.001) exhibited optimal efficacy in the evaluation of chronic musculoskeletal pain. This observed association between the effectiveness of a specific assessment tool and positive outcomes may be linked to the sensitivity and specificity of these instruments in evaluating knee osteoarthritis [48], a condition that demonstrated enhanced responsiveness to mind–body exercise interventions in the scrutinized studies. The WOMAC is specifically crafted and validated for gauging pain, stiffness, and function in individuals with knee and hip osteoarthritis [49]. Due to its specificity to this condition, it is presumably more sensitive in detecting significant alterations in pain and function within this population. Studies utilizing the WOMAC likely concentrated on knee osteoarthritis, elucidating the superior performance of this instrument in evaluating the impact of mind–body interventions on chronic pain. Conversely, the VAS, recognized for its widespread use and versatility in measuring pain intensity [50], although not specifically tailored for knee osteoarthritis, demonstrated the capacity to capture subjective pain intensity and manifested positive effects in reflecting an improved perception of pain among study participants.

Mind–body interventions embody an integrative therapeutic approach that recognizes the intricate connection between the mind and body in the context of health and wellness [18]. These practices are rooted in the concept that mental, emotional, and cognitive states can significantly influence physical health, and conversely, physical well-being can impact mental and emotional states [51]. Ranging from relaxation and breathing techniques to more structured forms of exercise such as yoga, Tai Chi, or meditation, these interventions aim not only to address physical symptoms but also to foster harmony and balance between the mental and physical aspects of the individual [52]. The results gleaned from the systematic review and meta-analysis furnish valuable insights into the efficacy of mind–body interventions in the treatment of chronic musculoskeletal pain. With the inclusion of 11 articles and the utilization of a random-effects model, prompted by the low heterogeneity and variability observed among the studies (I^2^ = 10%; Q-Value = 12.182 with 11 degrees of freedom and *p* = 0.350), the results substantially contribute to our understanding of the therapeutic potential of mind–body interventions for this prevalent and challenging health problem. The analysis revealed a medium but statistically significant effect size of mind–body interventions on pain (g = 0.400, IC del 95% = 0.268–0.532; *p* < 0.001). This finding suggests that, overall, these interventions have a positive impact on reductions in chronic musculoskeletal pain, which can be understood through several inter-related physiological and psychological mechanisms. These include (i) the reduction in stress and inflammatory responses: Mind–body practices, such as meditation, yoga, or Tai Chi, are associated with stress reduction and nervous system regulation, leading to a decrease in the release of stress hormones, such as cortisol. This stress reduction may mitigate the systemic inflammatory response, subsequently reducing the perceived intensity of pain [53]. (ii) The modulation of the central nervous system: Mind–body interventions may influence pain perception by modulating central nervous system activity. For instance, meditation has been associated with changes in brain connectivity and neuronal plasticity, potentially altering the brain’s interpretation and response to chronic pain [54]. (iii) Improvements in musculoskeletal function and posture: Exercises such as Tai Chi and yoga involve gentle movements, stretching, and muscle strengthening, leading to improved flexibility, strength, and posture. This, in turn, reduces the physical load on joints and muscles affected by chronic pain, potentially decreasing pain perception [55]. (iv) The stimulation of endorphin release: The regular practice of mind–body exercises can stimulate the release of endorphins, neurotransmitters associated with pain reduction, and mood improvement. This acts as a natural analgesic, decreasing the sensation of pain in individuals with chronic musculoskeletal pain [56].

Likewise, the findings presented offer crucial insights into the relative effectiveness of different mind–body interventions in managing chronic musculoskeletal pain. Notably, Tai Chi stands out compared to yoga and Qigong, demonstrating a more pronounced effect on pain reduction (g = 0.635, 95%CI = 0.311–0.959; *p* < 0.001). The distinctive nature of Tai Chi, combining gentle, flowing movements with breathing techniques and mindfulness approaches, may explain its greater impact on pain reduction. The holistic approach of Tai Chi likely contributes to a more comprehensive improvement in musculoskeletal function, flexibility, and posture. The complexity of its movements and emphasis on body alignment could reduce muscle tension, decrease the load on affected joints, and promote relaxation, potentially leading to a more significant reduction in the perception of chronic pain [57]. However, it is crucial to note that these results do not invalidate the efficacy of yoga or Qigong in managing chronic musculoskeletal pain. Both practices showed positive, albeit more modest effects compared to Tai Chi. The variability in intensity, sequence of movements, and mindfulness required in these practices could have influenced their effects on pain reduction [58]. Additionally, it is essential to consider that the effectiveness of these interventions may be influenced by individual factors, such as the patient’s prior experience with these practices, adherence to the program, as well as the severity and specific nature of the chronic pain [59].

The present review has both strengths and limitations. In terms of methodological quality, a significant limitation is that no intervention blinded therapists or participants, potentially introducing biases in result interpretation. Nevertheless, it is noteworthy that there is a low risk of publication bias, and the overall consistency in the methodological quality of the included studies is encouraging; however, the absence of successful blinding procedures for both participants and therapists across studies introduces a potential source of bias, which could result in an overestimation of the observed outcomes. On the other hand, the low heterogeneity and variability in the meta-analysis further enhances confidence in the results and their general applicability. A geographical imbalance is identified in the origin of the studies, with the majority conducted in Europe and America. Only one study each was conducted in Australia and Asia, and no research was found in Africa. This raises potential limitations in the generalizability of the findings, as cultural aspects, including beliefs, values, and norms, may impact people’s willingness to participate in and respond to mindfulness interventions. Moreover, the variability in access to mental health resources and services across regions may restrict the effective implementation of these interventions in specific areas. Addressing these limitations through more comprehensive and carefully controlled research could provide valuable insights into the effectiveness of mind–body interventions.

## 5. Conclusions

The current review underscores the potential benefits of mind–body exercise interventions in alleviating chronic musculoskeletal pain among older adults. The clinical implications derived from these findings are noteworthy, offering a potentially effective non-pharmacological therapeutic alternative that could significantly enhance the quality of life in this population. The research suggests that health professionals should seriously consider integrating mind–body exercise programs into the management of chronic pain in older adults, providing more holistic and complementary therapeutic options to existing conventional treatments. Furthermore, these results emphasize the importance of understanding and leveraging the interconnection between the mind and body in pain management, which may significantly influence the design of future therapeutic approaches to more comprehensively address these types of conditions.

## Figures and Tables

**Table 1:** Methodological quality of the included articles.

	1	2	3	4	5	6	7	8	9	10	11	Total Score
Liu et al., 2019 [30]	1	1	0	1	0	0	0	1	0	1	1	5
Morone, Greco, and Weiner, 2009 [31]	1	1	1	1	0	0	0	0	1	1	1	6
Morone et al., 2016 [32]	1	1	1	1	0	0	1	1	1	1	1	8
Cruz-Díaz et al., 2015 [33]	0	1	0	1	0	0	1	1	0	1	1	6
Yang, Kim and Lee, 2005 [34]	1	1	0	1	0	0	0	1	0	1	1	5
Teut et al., 2016 [35]	1	1	1	1	0	0	0	1	1	1	1	7
Fransen et al., 2007 [36]	1	1	1	1	0	0	1	1	1	1	1	8
von Trott et al., 2009 [37]	1	1	0	1	0	0	0	0	1	1	1	5
Cheung et al., 2014 [38]	1	1	1	1	0	0	1	1	1	1	1	8
Cheung et al., 2017 [39]	1	1	1	1	0	0	0	1	1	1	1	7
You et al., 2018 [40]	1	1	0	1	0	0	1	0	1	1	1	6

Items: 1 = eligibility criteria; 2 = random allocation; 3 = concealed allocation; 4 = baseline comparability; 5 = blind subjects; 6 = blind therapists; 7 = blind assessors; 8 = adequate follow-up; 9 = intention-to-treat analysis; 10 = between-group comparisons; 11 = point estimates and variability; Yes = 1; No = 0.

**Table 2:** Characteristics of the included studies.

Author and Year	Sex %	Condition and Pain Duration	Sample CG/IG	Control Group	Age	Intervention	Intervention Parameters	Results
Liu et al., 2019 [30]	F: 73.3 M: 26.7	Lower back pain > 3 months	13/15	Unaltered lifestyle	58.13 ± 5.38	Tai Chi	F: 3 times/week #S: 48 sessions D: 60 min	Tai Chi decreased pain compared to the control group (3.47 ± 0.99 vs. 5.85 ± 0.8, *p* < 0.01).
Morone, Greco, and Weiner, 2009 [31]	F: 52.6 M: 47.4	Lower back pain > 3 months	13/12	Wait list	74.1 ± 6.1	Mindfulness and meditation	F: 1 time/week #S: 8 sessions D: 90 min	Compared to the control group, the intervention group displayed significant improvement in the Chronic Pain Acceptance Questionnaire Total Score and Activities Engagement subscale (*p* = 0.008, *p* = 0.004).
Morone et al., 2016 [32]	F: 66.4 M: 33.6	Lower back pain > 3 months	142/140	10 Keys to Healthy Aging program	75 ± 7.2	Mindfulness-based stress reduction program	F: 1 time/week #S: 8 sessions D: 90 min	By 6 months, the intervention participants improved on the Numeric Pain Rating Scale in current and most severe pain measures by an additional −1.8 points (95% CI, −3.1 to −0.05 points; effect size, −0.33).
Cruz-Díaz et al., 2015 [33]	F: 100	Lower back pain > 3 months	50/47	Physiotherapy-only group	71.14 ± 3.30	Pilates + physiotherapy	F: 3 times/week #S: 18 sessions D: 60 min	Pilates combined with physiotherapy had a substantial and statistically significant mean effect on pain in women with chronic lower back pain (d = 1.46).
Yang, Kim, and Lee, 2005 [34]	F: 68 M: 32	General chronic pain > 3 months	21/19	Wait list	72.58 ± 5.41	Qi-therapy	F: 2 times/week #S: 8 sessions D: 20 min	Compared with baselinevalues, pain and psychological benefits remained significantly improved aftertwo weeks of follow-up (<0.001).
Teut et al., 2016 [35]	F: 88.5 M: 11.5	Lower back pain > 6 months	57/61	Wait list	73.0 ± 5.6	Yoga	F: 2 times/week #S: 24 sessions D: 45 min	The mean adjusted pain intensity after 3 months was 1.71 for the yoga group (95% confidence interval [CI], 1.54–1.89), 1.67 for the Qigong group (95% CI, 1.45–1.89), and 1.89 for no intervention (95% CI, 1.67–2.11). No statistically significant group differences were observed.
	F: 86.2 M: 13.8		57/58		72.4 ± 5.7	Qigong	F: 1 time/week #S: 12 sessions D: 90 min	
Fransen et al., 2007 [36]	F: 68 M: 32	Hip and knee osteoarthritis > 12 months	41/56	Wait list	70.8 ± 6.3	Tai Chi	F: 2 times/week #S: 24 sessions D: 60 min	After 12 weeks of practicing Tai Chi, it was shown to be effective in reducing pain in individuals with hip and knee osteoarthritis.
von Trott et al., 2009 [37]	F: 95 M: 5	Chronic neck pain > 6 months	40/38	Wait list	75.9 ± 7.6	Qigong	F: 2 times/week #S: 24 sessions D: 45 min	No significance difference between Qigong group and waiting list (47.4 ± 30.8 vs. 54.9 ± 28.5, *p* = 0.100)
Cheung et al., 2014 [38]	F: 100%	Knee osteoarthritis > 6 months	18/18	Wait list	71.9 ± 3.5	Yoga	F: 1 time/week #S: 8 sessions D: 60 min	Based on ANCOVAs, participants in the treatment group exhibited significantly greater improvement in WOMAC pain (Mean Diff = 8.3 ± 0.67; *p* = 0.010), stiffness and SPPB.
Cheung et al., 2017 [39]	F: 84% M: 16%	Knee osteoarthritis > 6 months	13/32	Education control	68.9 ± 7.7	Hatha yoga	F: 3–5 times/week #S: 24–40 sessions D: 45 min	After 8 weeks of practicing Hatha yoga, it was shown to be effective in reducing pain in individuals with knee osteoarthritis (WOMAC Post: 26.4 [22.5, 30.2], *p* = 0.001).
You et al., 2018 [40]	F: 72.73% M: 27.27%	Multisite pain > 3 months	23/22	Light physicalexercise group	74.27 ± 7.48	Tai Chi	F: 2 times/week #S: 24 sessions D: 60 min	Tai Chi significantly lowered pain severity (Pre: 4.58 ± 1.73; Post: 3.73 ± 1.79; *p* < 0.01) and pain interference (Pre: 4.20 ± 2.53; Post: 3.16 ± 2.28; *p* < 0.05).

F: frequency; #S: number of sessions; D: duration; CG: control group; IG: intervention group; WOMAC: Western Ontario and McMaster.

## Data Availability

Not applicable.
